# Abdominal Surgery

**Published:** 1898-06

**Authors:** 


**Abdominal Surgery.** By J. Greig Smith. Sixth Edition. Edited by James Swain, M.S., M.D. Two vols. Pp. xiii., 1—576, xii.; x., 577—1171, xxvii. Bristol: J. W. Arrowsmith. 1897.

The fifth edition of this work was published in 1896, and within a year it became necessary to issue a sixth. The author requested his colleague, Dr James Swain, to relieve him of the labour of bringing out the new edition, scarcely a hundred pages of which had been prepared when the master-hand was called away and the remainder of the work was entirely undertaken by Dr. Swain. We have little doubt that as it now stands the book will remain for many years the standard text-book of abdominal surgery. Advances in this subject are doubtless continually being made, and year by year we see alterations and improvements in diagnosis and technic, but the broad principles here enunciated will certainly remain as a memorial of the earnest worker and original thinker who has brought them into a compact form so easy of reference.

The early sections are devoted to the subject of abdominal operations generally considered. The author was one of the first to point out that ovariotomies and other delicate abdominal operations could be successfully performed in general operating theatres. He acknowledges that there is an *a priori* probability of the greatest success being obtained in specially designed hospitals fully under the control of one surgeon, but he considers that extra care in avoiding all matters conducive to septicism justify their being carried out either in general hospitals or in private dwellings.

The section on ovariotomy is one of the best in the book. An interesting account is given of the early history of the operation. Due credit is given to Ephraim McDowell as the first ovariotomist, and the claims of Houston of Glasgow are at once put aside. The labours of other American surgeons to improve the operation are duly acknowledged, while the work of Spencer Wells, Thomas Keith, and Knowsley Thornton receives full recognition.

The chapters treating of operations for ectopic gestation have been carefully brought up to date. The author holds with Lawson Tait that all extra-uterine conceptions are at first tubal, and that further varieties are produced after rupture of the primary tubal sac and escape of the ovum. He urges early and prompt operation in almost all cases. He points out that treatment by electricity is only suitable in the early stages, but it is not always successful, it is not free from danger, and, in its limited application, it enters into competition with cœliotomy in the same field where the latter is most successful in primary results, and has also secondary results which are absolutely perfect.

Section VI. treats of operations on the stomach, and in the account of the operation of gastrostomy the author very wisely does not attempt to describe all the numerous modifications of the operation which have been brought forward from time to time by different surgeons, but has contented himself with describing fully the operation he himself preferred and has then given a short account of three other methods, viz., Witzel's, Frank's, and Paul's. Greig Smith had himself met with a somewhat unusual amount of success in his operations of gastrostomy, and his description of the details of his method and his simple means of avoiding leaking of the aperture are worthy of close study. It is very doubtful if any of the complicated methods often advocated are any improvement upon the simple procedure here recommended.

Operations on the intestine are fully dealt with, and the different means we possess of uniting divided bowel are copiously illustrated. The author was never very partial to the Murphy button, but preferred a method by simple suture wherever the bowel was sufficiently healthy and the patient in good condition. Excellent descriptions are given of the best means of clamping and holding the bowel and of inserting the sutures, and sound rules are formulated for choosing a particular operation for a particular case.

We naturally look to the chapters on the general principles for operation in cases of intestinal obstruction, for the author had well-known and strong views on the best methods of dealing with these cases. He was a great advocate for endeavouring, before all things, to save the patient's life before attempting any prolonged operation which might seem theoretically desirable. To this end he urges that in many cases the early treatment should be by incision of bowel and evacuation of contents, the open bowel being afterwards fixed in the wound and a drainage tube inserted. This simple operation which may, if necessary, be performed without an anæsthetic was the means in the author's hands of saving many lives, and he strongly advocates its adoption in cases where the patient's strength is not equal to a more serious operation.

The sections on the surgery of the kidneys, and on that of the liver and gall-bladder, are among the best in the book, and are chiefly notable as being terse and didactic, so as to form an excellent guide for a young surgeon wishing for a trustworthy authority. The work of Lawson Tait in these fields is fully acknowledged, while at the same time the author draws freely upon his own very considerable experience.

The final section deals with the subject of peritonitis. The author makes a good working classification into simple, septic, and specific. He speaks favourably of the use of purgatives in this affection and does not altogether condemn the cautious administration of opium, but he puts aside belladonna as at best useless and at worst harmful.

Sub-phrenic abscess is fully described and directions are given for operating on this obscure and dangerous malady, while pelvic cellulitis and tubercular peritonitis are more briefly but effectively dealt with.

The book as a whole is characterised by the individuality of the author, whose strength of mind dominates all he has to say. It is evidently the work of one who had tried and thought out the methods recommended. It is no mere compilation, but is written by a worker for workers. As we turn over its pages we are filled with regret that the author's work should have terminated so prematurely and that he was not spared to bring out this sixth edition. Our readers will agree with us, however, that the editorship has been ably carried out by Dr. Swain, who for many years had assisted Greig Smith in his abdominal work.

